# Pain Measurement through Temperature Changes in Children Undergoing Dental Extractions

**DOI:** 10.1155/2016/4372617

**Published:** 2016-04-26

**Authors:** Eleazar S. Kolosovas-Machuca, Mario A. Martínez-Jiménez, José L. Ramírez-GarcíaLuna, Francisco J. González, Amaury J. Pozos-Guillen, Nadia P. Campos-Lara, Mauricio Pierdant-Perez

**Affiliations:** ^1^Coordinación para la Innovación y Aplicación de la Ciencia y la Tecnología, Universidad Autónoma de San Luis Potosí, Avenida Sierra Leona 550, 78210 San Luis Potosí, SLP, Mexico; ^2^Departamento de Epidemiología Clínica, Facultad de Medicina, Universidad Autónoma de San Luis Potosí, Avenida Venustiano Carranza 2405, 78210 San Luis Potosí, SLP, Mexico; ^3^Posgrado de Estomatología Pediátrica, Facultad de Estomatología, Universidad Autónoma de San Luis Potosí, Avenida Manuel Nava 4, 78290 San Luis Potosí, SLP, Mexico

## Abstract

*Background and Objective. *Pain evaluation in children can be a difficult task, since it possesses sensory and affective components that are often hard to discriminate. Infrared thermography has previously been used as a diagnostic tool for pain detection in animals; therefore, the aim of this study was to assess the presence of temperature changes during dental extractions and to evaluate its correlation with heart rate changes as markers of pain and discomfort.* Methods*. Thermographic changes in the lacrimal caruncle and heart rate measurements were recorded in healthy children scheduled for dental extraction before and during the procedure and compared. Afterwards, correlation between temperature and heart rate was assessed.* Results*. We found significant differences in temperature and heart rate before the procedure and during the dental extraction (mean difference 4.07°C, *p* < 0.001, and 18.11 beats per minute, *p* < 0.001) and no evidence of correlation between both measurements.* Conclusion*. Thermographic changes in the lacrimal caruncle can be detected in patients who undergo dental extractions. These changes appear to be stable throughout time and to possess very little intersubject variation, thus making them a candidate for a surrogate marker of pain and discomfort. Future studies should be performed to confirm this claim.

## 1. Introduction

Pain evaluation in small children can be a difficult task, since it is a multidimensional experience that possesses sensory and affective components that are often hard to discriminate by the existing scores [1, 2]. Previous experiences, fear, anxiety, and discomfort may alter pain perception; thus, poor agreement between different instruments and different raters is often the norm [3, 4]. It has been suggested that, in children younger than 7 years of age and in cognitively impaired children, evaluation of pain intensity through self-report instruments can be inaccurate due to poor understanding of the instrument and poor capacity to translate the painful experience into verbal language; therefore, complementary observational pain measurements should be used to assess pain intensity [4]. Observational pain measurements focus on behavioral clues, such as facial expressions, movement, cry, and sleeping cycles, that allow identification of the presence of pain and its quantification in a qualitative basis [2, 4, 5]. These measurements have been shown to possess higher degrees of agreement between raters and among different instruments [4] and can be complimented with surrogate markers based on physiological changes that occur in response to pain, like heart rate changes and cortisol serum levels [6, 7]. Although these markers have high degree of intra- and intersubject variability, they have been included in some observational pain measurements scores [4, 8].

Infrared thermography has been used for the evaluation of temperature distribution in different anatomical locations [9], as well as a diagnostic and therapeutic tool to guide treatment in diseases such as neuropathic pain, dental pulp pathology, complex regional pain syndromes, postherpetic neuralgia, whiplash injuries, inflammatory arthritis, temporomandibular joint disorders, headache, and myofascial pain syndromes [10–13]. In animals, thermography has demonstrated high sensitivity for the detection of pain through changes in the skin temperature distribution due to sympathetic pain induced responses and a high degree of correlation between the severity of pain and the thermographic dermal changes [14].

Dental extractions are one of the most painful and discomforting treatments that are performed in otherwise healthy children, even though they are anesthetized. Since these children are often impaired to communicate their pain through verbal clues because of the procedure and observational measurements may be difficult to monitor throughout the surgery, there is a need of surrogate markers for pain evaluation. The aim of this study was to evaluate temperature changes, as measured by infrared thermography during tooth extractions, and to evaluate their correlation with heart rate changes as markers of pain and discomfort. We hypothesized that both variables would present differences in measurements done before and during the procedure and that there would be a correlation between heart rate and temperature throughout time.

## 2. Patients and Methods

### 2.1. Patients

The study was performed in 18 healthy children (13 boys and 7 girls; mean age 9.4 ± 1.6 years) scheduled for extraction of one first deciduous molar because of orthodontic reasons in the Pediatric Dentistry Clinic of Universidad Autónoma de San Luis Potosí, Mexico. Tooth extraction was performed under regional anesthesia using the conventional technique, either in the maxilla (*n* = 11) or in the mandible (*n* = 7). The study was approved by the University's Ethics Committee (registry CEIFE-005-010), and all parents provided their informed consent.

### 2.2. Measurements

**Figure 1:**
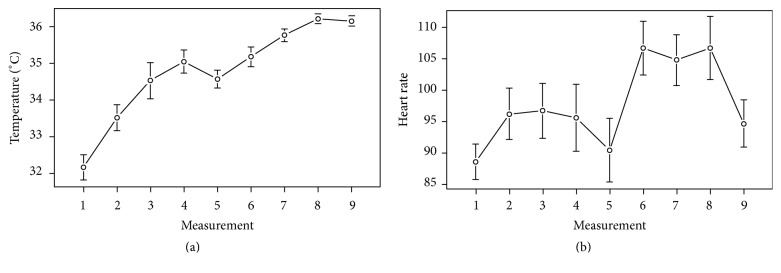
Heart rate and thermographic measurements. Heart rate (a) and thermographic measurements (b) were taken at 1-minute intervals before starting the procedure (measurement 1), during the application of the anesthesia (measurements 2 to 5), during the tooth extraction (measurements 6 to 8), and after the extraction (measurement 9). Bars indicate 95% confidence intervals.

**Figure 2:**
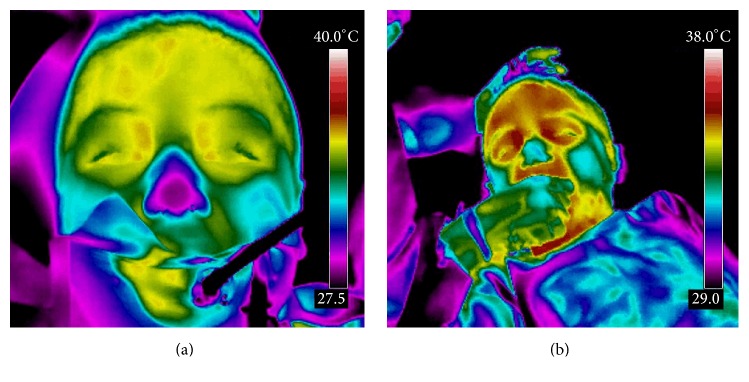
Infrared thermographic scan. A thermographic scan of children's face before (a) and during (b) the procedure is shown. Both medial canthi are easily observed as “hot” spots in the first panel, and an area of skin response is evident during the extraction. Measurements for the present study were taken ipsilateral to the side of the tooth extraction.

Heart rate (beats per minute, bpm) and thermographic measurements in degrees Celsius (°C) were recorded at approximately 1-minute intervals before starting the procedure (measurement 1, basal), during and after the children had been anesthetized (measurements 2 to 5, presurgical measurements), during tooth extraction (measurements 6 to 8, trans-surgical measurements; measurement 8 was the moment of tooth extraction), and after the procedure (measurement 9, postprocedure) (Figure 1). To evaluate heart rate, a pulse oximeter (NT1 Handheld Pulse Oximeter, Newtech Inc., Guangdong, China) was placed at the end of the index finger. Pulse oximetry measures the capillary bed blood flow in the finger, thus detecting heart rate. Thermal measurement was done through infrared imaging with a FLIR T400 infrared camera (FLIR Systems, Wilsonville, OR) with a 320 × 240 focal plane array of uncooled microbolometers with a spectral range of 7.5 to 13 lm and a thermal sensitivity of 50 mK at 30°C in the lacrimal caruncle of the right eye, since this area has previously been shown to be a site of dynamic temperature changes in response to pain [14, 15]. Thermographic data analysis was performed using FLIR QuickReport v.1.2 (FLIR Systems Inc., North Billerica, MA), which includes a tool to obtain maximum, minimum, and average temperature of a user-defined area (Figure 2).

### 2.3. Statistical Analysis

Statistical analysis was carried out using the statistical package R v.3. 1. 2. Results are expressed as mean ± SD. The assumption of normal distribution of the data was evaluated and confirmed through Shapiro-Wilk tests, and a parametric analysis was performed with paired Student's *t*-test between measurements performed during time 1 (basal measurement) and time 8 (moment of tooth extraction). Correlation between temperature and heart rate at these times was assessed by a linear regression model; and to assess the correlation of measurements throughout time, mixed effects repeated measures model was performed. A value of *p* ≤ 0.05 was considered to be statistically significant.

## 3. Results

**Figure 3:**
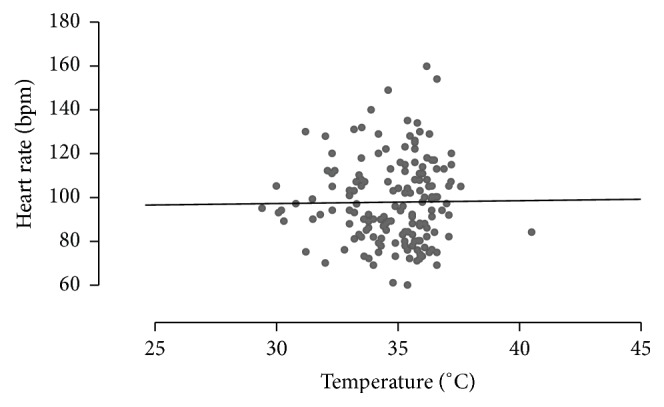
Correlation between temperature and heart rate. The scatterplot shows the relation between temperature and heart rate for all measurements. We found no correlation between them (*p* = 0.46).

Difference in temperature before the procedure (measurement 1, basal) and at the moment of the dental extraction (measurement 8) was 4.07°C (95% CI: 3.27–4.87; *p* < 0.001). Difference in heart rate was 18.11 bpm (95% CI: 10.35–25.87; *p* < 0.001) (Table 1). We found no evidence of correlation between temperature and heart rate at the moment of the first measure (*R* ^2^ = 0.16; *p* = 0.10), at the moment of the tooth extraction (*R* ^2^ = 0.02; *p* = 0.57), or in the mixed effects repeated measures model (*p* = 0.46) (Figure 3).

## 4. Discussion

Pain severity in children undergoing procedures can be difficult to assess, since they feel anxiety even before pain is perceived; and anxiety may bias pain intensity measurement by clinical scores [12]. Because proper handling of pain is a priority in the postoperative care [16], studies that aim to improve its diagnosis through the use of innovative techniques, such as thermography, will surely have a great impact in the near future as tools to measure the effectiveness of pain treatment.

Infrared thermography (IRT) detects infrared light (heat) emitted by the body. Changes in the amount of body heat are due to changes in the skin's blood flow. Thus, skin thermography can be used as a tool for detecting pain because this organ is controlled by the combined effects of the central and the autonomic nerve system (ANS) for temperature regulation, and it is prone to changes in temperature distribution in response to ANS stimulation. IRT is not a tool that shows anatomical abnormalities. It is a technique that shows physiological changes in the organ [12].

Acute physiological responses to noxious stimuli include activation of the ANS and hypothalamic-pituitary-adrenal axis, since these systems mediate a stress response and restore the metabolic homeostasis in tissues. Acute pain and anxious states activate the sympathetic division of the ANS to release catecholamines from the adrenal medulla as part of a quick “fight or flight” reaction. In contrast, cortisol mediated responses originated in the hypothalamic-pituitary-adrenal axis have slower onsets, are more persistent, and can be measured as increment in the serum cortisol levels. Several physiological variables, such as heart rate, eye pupil diameter, skin temperature, peripheral blood flow, and plasma catecholamine concentrations, can be used to measure the amount of sympathetic nervous system (SNS) activity. Catecholamines promote energy mobilization, blood vessel dilation, and increased muscle contractility, altering cardiac output, respiratory rate, and other responses required for the rapid “fight or flight” response [17, 18].

Pain evaluation through changes in eye temperature has been previously examined in cattle [14, 15] and in patients with neuronal ceroid lipofuscinosis [19]. It has been suggested that eye's temperature measured on the lacrimal caruncle, which estimates the capillary blood flow of the conjunctival bed, when measured by infrared thermography may be a practical and noninvasive measurement of SNS activity [17]. An increase in the lacrimal caruncle's temperature after a painful procedure can be attributed to a sympathetically mediated alteration in the blood flow, even when the painful stimuli were evoked away from the measured area [14]. Since the thermal response seen on our patients concurs with these observations, we believe that it is also due to SNS pain induced activity. Moreover, intersubject variation seems to be small, which may rule out that nerve irritation during anesthesia could act as a confounding factor, since several children had teeth extracted from the mandible.

Our results suggest that thermographic measurements can be used as surrogate markers of pain and discomfort in children undergoing dental procedures, since we found significant differences between measurements taken before the procedure and during the dental extraction. More importantly, the trend of these measurements appears to be more stable throughout time than measurements of heart rate, which have traditionally been used as a surrogate marker of pain. Although we did not find correlation between measurements, a trend between heart rate and temperature is evident in Figure 1, and temperature changes are much stable throughout time, as evidenced by the slope of the graph, and they have very little intersubject variability, as evidenced by their small confidence intervals. Heart rate, on the other side, has a great degree of variation throughout time and between subjects, as evidenced by the wide confidence intervals of the measurements and the wide variations observed at different times. We believe that this was the main reason for not finding correlation between measurements: while both measurements follow a similar trend (both rise during the anesthetization, fall in time 5 after anesthesia was administered, and increase once again during the procedure), heart rate is a very dynamic physiological variable. Temperature changes are a more stable response to SNS stimulation.

Several pain measurement scores for clinical use in children have been proposed. The most simple are visual analogue scores, which use colors or faces that measure self-reported pain intensity. These scores have the disadvantage of being unreliable in small children because they tend to report “how they feel” and not how much pain they feel [20, 21]. Observational scores, while robust for the assessment of pain in small or cognitively impaired children, have the disadvantage that a period of observation is needed before measurements are performed, which may complicate the assessment and treatment of acute pain. Surrogate markers of pain are the only measurements able to provide a real-time feedback on pain intensity [2]. Thermometric changes may act as a surrogate marker for pain to complement algometry in children. This method has the advantages of being noninvasive, that it can be measured in real time, and that it does not require the children to lay immobile for a prolonged period of time, since the infrared camera image acquiring is analogue to the acquiring of images from a conventional digital camera.

The main limitation of this study was the fact that temperature and heart rate measurements were not compared against a control group, which may have allowed us to determine if some amount of change could be due to anxiety and not pain. Also, we did not compare our measurements against a self-reported pain score, since in dentistry these scores need to be applied after the procedure, which could introduce recall bias into the result.

In conclusion, thermographic changes in the lacrimal caruncle can be used to detect pain in patients undergoing dental extractions. These changes appear to be stable throughout time and possess very little intersubject variation, thus making them a candidate for a surrogate marker of pain and discomfort. Temperature recording through thermographic scans may be used as a physiological measurement of pain to complement current scores. Future studies should be performed to confirm these claims.

## Figures and Tables

**Table 1:** Difference in heart rate and temperature before the procedure and during dental extraction.

	Time 1	Time 8	*p*
Heart rate, bpm	88.61 ± 11.90	106.72 ± 15.97	*p* < 0.001
Temperature, °C	32.16° ± 1.46	36.23° ± 0.59	*p* < 0.001

^*∗*^Data are presented as mean ± SD. A paired Student's *t*-test was used for comparisons. bpm = beats per minute; °C = degrees Celsius.

## Abbreviations

°C: Degrees Celsius
ANS: Autonomic nerve system
bpm: Beats per minute
IRT: Infrared thermography
SNS: Sympathetic nervous system.

## Additional Points

Pain evaluation in children can be a difficult task, since it possesses sensory and affective components that are hard to discriminate. We used infrared thermography to assess the presence of temperature changes during dental extractions in healthy children and to evaluate its correlation with heart rate as markers of pain and discomfort. We found significant differences in both measurements and no evidence of correlation between them. Thermographic changes appear to be stable throughout time and to possess very little intersubject variation, thus making them a candidate for a surrogate marker of pain and discomfort in children.
